# Decoding the Digital Discourse: A Thematic and Sentiment Analysis of Reddit Posts on Eyelid Surgery

**DOI:** 10.1093/asjof/ojag050

**Published:** 2026-03-18

**Authors:** Henry Bair, Haley Evans, Lara Cheslow, Charlotte L Marous

## Abstract

**Background:**

Eyelid surgery is among the most performed aesthetic and functional procedures, yet existing patient-reported outcome measures may miss unprompted concerns voiced on social media.

**Objectives:**

The aim of this study was to characterize emotions, language use, and patient priorities in Reddit discussions about eyelid surgery.

**Methods:**

The authors performed a cross-sectional mixed-methods analysis of English-language posts and top-level comments from 8 subreddits (May 12, 2024 to May 12, 2025). A transformer-based model and the National Research Council Emotion Lexicon were used to assign sentiment polarity and discrete emotions and to quantify postcomment concordance. Sociolinguistic features (lexical richness, readability, and medical terminology density) were computed for all entries. A random sample of 500 entries underwent inductive thematic analysis.

**Results:**

The authors analyzed 1579 posts and 3576 top-level comments. Posts demonstrated mixed sentiment polarity (39.0% positive, 32.2% negative, and 28.8% neutral), whereas comments were more often positive (55.8%) than negative (24.1%) or neutral (20.1%). Across postcomment pairs (1 pair per comment), 64.5% were discordant, most commonly negative post → positive comment. Fear and sadness predominated in posts, whereas trust and anticipation were more common in comments. Medical terminology appeared frequently yet was often imprecise. Thematic analysis yielded 9 themes, including preoperative anxiety, dissatisfaction despite acceptable results, distress over mild asymmetry, and social-media-driven surgeon selection.

**Conclusions:**

Reddit discourse reveals clinically literate but diagnostically uncertain patients who judge success by culturally inflected, photo-salient aesthetics and rely on peer reassurance. Early vocabulary alignment and explicit counseling on asymmetry and variability may improve expectation setting and generate testable hypotheses for counseling strategies.

**Level of Evidence: 5 (Therapeutic):**

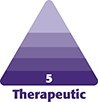

Blepharoplasty is among the most frequently performed cosmetic surgeries worldwide.^1,2^ Eyelid surgery is performed not only to improve a more alert and youthful appearance but also to correct visual field obstruction and lid asymmetry. Patient motivations are often deeply personal, shaped by anatomical concerns as well as psychological, social, and cultural influences.^3,4^ Validated patient-reported outcome measures (PROMs), such as the FACE-Q Eye Module, exist and capture satisfaction, psychosocial well-being, and decision quality after blepharoplasty; however, outcomes reporting across practices remain heterogeneous, limiting cross-study comparability, and leaving important nuances of the patient experience underdescribed.^5^

In recent years, social media has emerged as a dominant source of medical information, with up to 90% of all patients reportedly turning to platforms like Facebook, Twitter, and Instagram for health-related education, inspiration, and emotional support.^6,7^ Reddit offers a distinct format: anonymous, discussion-based communities where users share their insecurities, surgical decisions, and recovery journeys. These unfiltered conversations offer rich, underexplored insight into what patients worry about, expect, and value most.^8^

Several medical specialties, including ophthalmology, have leveraged Reddit to analyze patient concerns and emotional sentiment, using these insights to inform perioperative care.^9-12^ A previous study characterized the types of general ophthalmology questions patients posited on Reddit, showing that posts frequently revolve around clinical concerns but that comments only rarely recommend seeking professional care, highlighting both demand for guidance and the risk of misinformation.^13^

Beyond Reddit, sentiment analyses of patient conversations around oculoplastics on web forums demonstrate that negative emotions, especially fear and sadness, are common around blepharoplasty, but these studies do not integrate qualitative thematic analysis to explain why those emotions arise.^14^

To address this gap, we analyzed public Reddit discussions related to eyelid procedures to characterize emotional valence and specific emotions in posts vs comments, map recurring psychosocial themes, and evaluate how lay and medical terminology are used. By centering the unprompted “patient voice” that increasingly shapes expectations before the clinic, our goal is to inform surgeon–patient communication, sharpen preoperative counseling, and support shared decision making for both functional and aesthetic eyelid surgery.

## METHODS

### Study Design and Reporting

We performed a cross-sectional, mixed-methods analysis of public Reddit discussions on eyelid procedures. Reddit was selected as the primary data source because of its broad user base, accessibility, and high volume of health-related discussions, including cosmetic and reconstructive surgery. Quantitative natural language processing (NLP) analyses included sentiment polarity, postcomment concordance, and sociolinguistic features; the qualitative strand used inductive, reflexive thematic analysis. The study used only public, pseudonymous content without interaction. In line with internet research ethics guidance, we minimized traceability (no usernames; quotes paraphrased where necessary), removed personally identifying details, and aggregated findings at the theme level. This study, which relied on retrospective review of publicly available anonymous data, was exempt from IRB. Reporting follows the Standards for Reporting Qualitative Research and the Good Reporting of a Mixed-Methods Study (GRAMMS) recommendations.^15-17^

### Data Source, Extraction, and Preprocessing

Data were extracted using the Python Reddit API Wrapper (PRAW, Python 3.12), which allows for structured programmatic access to public Reddit content. We queried 8 subreddits: r/PlasticSurgery, r/AskDocs, r/HoodedEyes, r/Ophthalmology, r/TwoXChromosomes, r/SkincareAddiction, r/30PlusSkinCare, and r/EyeTriage. These communities were selected in advance of data extraction to minimize post hoc selection bias and because they collectively represent the major Reddit forums where individuals discuss eyelid appearance, ptosis, dermatochalasis, and periocular surgery. r/PlasticSurgery and r/HoodedEyes capture cosmetic and aesthetic concerns; r/AskDocs, r/EyeTriage, and r/Ophthalmology capture medically oriented presentations of eyelid symptoms; and r/TwoXChromosomes, r/SkincareAddiction, and r/30PlusSkinCare reflect broader demographic groups in which eyelid-related concerns frequently arise. Within each subreddit, we searched titles and bodies using clinical and colloquial terms related to eyelid surgery (eg, “ptosis,” “blepharoplasty,” “dermatochalasis,” “eyelid surgery,” “eyelid asymmetry,” “droopy eyelid,” “hooded eyes,” and “double eyelid surgery”).

Inclusion criteria were English-language posts between May 12, 2024 and May 12, 2025 that contained ≥1 keyword and ≥10 alphanumeric characters of user-generated text. Exclusion criteria were moderator/bot messages (including automoderator), posts/comments marked “[deleted]” or “[removed],” nontext content without accompanying text (eg, links only), duplicates, and cross-posts when an original was already included. For each eligible post, we collected the title, body, subreddit, UTC timestamp, and the top 5 top-level comments ranked by Reddit's default “Best” relevance algorithm; if <5 top-level comments were available, all available comments were included. Replies to comments (nested threads) were excluded.

Following extraction, we applied a multi-step cleaning protocol using Python (v3.12). Duplicate posts were removed. Text fields were normalized by stripping whitespace, converting to lowercase, and removing newline characters. Noninformative content—including posts and comments labeled as “[deleted]” or “[removed]” or identified as moderator generated—was excluded using keyword-based filters. Only user-generated entries containing substantive text were retained.

### Sentiment Polarity and Emotion Profiling

We applied a transformer-based NLP model trained on the GoEmotions framework to assign (1) sentiment polarity (positive/neutral/negative) and (2) a dominant emotion label to each Reddit post and each extracted top-level comment. GoEmotions is a widely used, publicly available emotion classification scheme developed from a large set of human-labeled short texts, in which each text is assigned 1 or more everyday emotion categories (eg, “gratitude,” “anxiety/nervousness,” “disappointment,” and “anger”) and a corresponding overall sentiment polarity. Each post and comment was analyzed independently. For polarity analyses, we used the model's sentiment label (positive/neutral/negative). For emotion analyses, we used the model's dominant emotion (the top-ranked label). For postcomment concordance analyses, each top-level comment was paired with its parent post (1 pair per comment). Concordance was defined as identical sentiment labels; discordance was defined as differing labels. We also tabulated the distribution of discordant transition types (eg, negative post → positive comment).

We performed sensitivity analyses by repeating key polarity estimates using alternative sentiment outputs generated during the same analytic pipeline (eg, multi-label sentiment labeling and rule-based override labeling) and compared these with the primary GoEmotions dominant-sentiment results.

As a nonparametric triangulation, we mapped tokens to the National Research Council (NRC) Emotion Lexicon, a widely validated, rule-based dictionary that maps over 14,000 English words to 8 primary emotions (anger, fear, anticipation, trust, surprise, sadness, joy, and disgust). We then compared aggregated emotion patterns for posts vs comments.^18^

### Sociolinguistic Measures

To assess how patients articulated their experiences, we examined 3 linguistic dimensions of the text. Lexical richness was estimated for each post and comment using the type–token ratio, a measure of vocabulary diversity. Readability was evaluated with the Flesch Reading Ease and the Flesch–Kincaid Grade Level indices, with higher grade levels reflecting more complex language. Finally, we quantified medical terminology density by scanning each entry against a curated list of 100 oculoplastic-specific terms encompassing diagnoses, anatomical structures, procedures, and perioperative care. This lexicon was drafted by the senior oculoplastic surgeon (C.L.M.) and refined through pilot coding. Term counts were normalized by total word count to enable comparison across entries of different lengths.

### Thematic Analysis

We used an inductive, reflexive thematic analysis to characterize patient concerns, expectations, and psychosocial contexts. Thematic analysis was conducted using NVivo (version 15; Lumivero, Denver, CO) to systematically identify recurring concerns, motivations, expectations, and emotional themes in patient discourse. Relevant fields—including post titles, body text, and top-level comments—were imported into NVivo for qualitative coding.

A simple random sample of 500 entries (posts plus their associated top-level comments), stratified 1:1 by (post vs comment), was selected using Python's random number generator with a fixed seed for reproducibility and exported to NVivo for qualitative coding. Excerpts of text were coded into thematic nodes representing recurring ideas (eg, “fear of asymmetry,” “insurance frustration,” “postoperative regret,” and “social media influence”). Nodes were then iteratively refined and grouped into broader thematic domains, such as preoperative concerns, postoperative outcomes, emotional responses, and systemic barriers. Coding discrepancies were resolved through discussion until consensus was reached.

A finalized thematic map was constructed by consolidating subthemes into overarching categories. Themes are reported with concise definitions and exemplar quotations labeled by subreddit and month/year; relative salience is conveyed qualitatively (high/medium/low-medium) rather than by numeric prevalence. These salience labels were assigned a priori to convey recurrence without implying statistical prevalence: high indicated subthemes appearing repeatedly across multiple posts and/or subreddits; medium indicated recurring but less widespread subthemes; and low-medium indicated episodic subthemes that nonetheless added conceptual or clinical nuance.

### Outcomes and Integration

Primary quantitative outcomes were (1) polarity distributions for posts and for comments, (2) postcomment concordance across all pairs, with a breakdown of discordant types (eg, negative post → positive comment), (3) lexical richness and readability of the text, and (4) prevalence of medical terminology. Qualitative outcomes were the final theme set and subthemes with definitions and representative quotations.

Per GRAMMS, we integrated strands at interpretation: (1) Convergence: Do model- and lexicon-based emotions align with the valence observed in themes? (2) Complementarity: Do themes explain why particular emotions cluster around specific decision points (eg, preoperative evaluation vs early recovery)? (3) Expansion: Do rare but clinically important concerns emerge qualitatively despite low frequency quantitatively?^15^ Integration products include joint displays linking emotions, themes, and exemplar quotations.

Analyses were primarily descriptive (counts, percentages, and medians where relevant); we additionally used χ^2^ tests to compare sentiment-category distributions between posts and comments, with 2-sided *α* = .05 defining statistical significance.

## RESULTS

### Sentiment, Emotional, and Lexical Analysis

**Figure 1.**
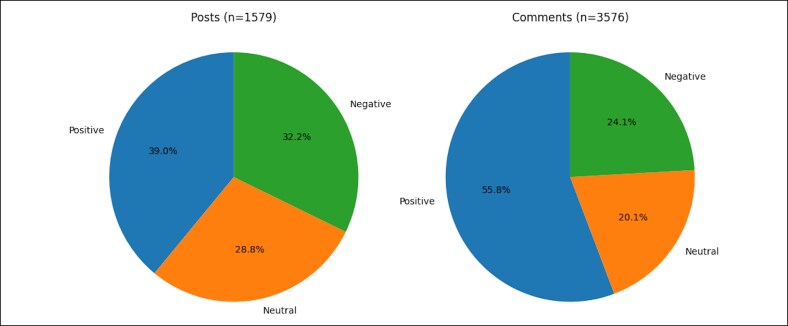
Sentiment polarity distribution in Reddit posts vs comments. Pie charts show the proportion of transformer-assigned sentiment labels (positive, neutral, and negative) among posts (*n* = 1579) and top-level comments (*n* = 3576) across eyelid surgery–related Reddit discussions.

**Figure 2.**
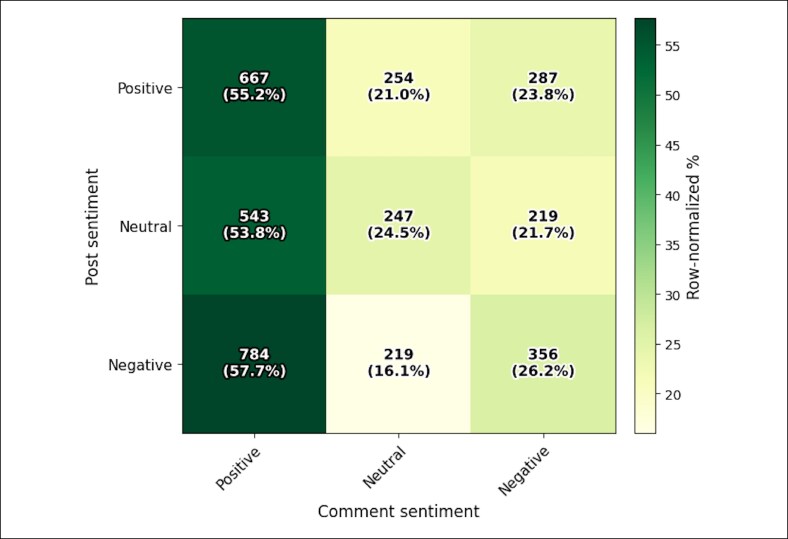
Postcomment sentiment discordance in Reddit eyelid surgery discussions. The heat map displays the distribution of comment sentiment conditional on parent postsentiment (row-normalized percentages), with each top-level comment paired to its corresponding post (1 pair per comment; *n* = 3576). Cell annotations indicate count and row percentage; discordant transitions reflect sentiment mismatch between posts and replies.

A total of 1579 posts and 3576 comments were analyzed. Posts were positive (616/1579, 39.0%; 95% CI, 36.6%-41.4%), negative (509/1579, 32.2%; 95% CI, 30.0%-34.6%), and neutral (454/1579, 28.8%; 95% CI, 26.6%-31.0%). Comments were more often positive (1994/3576, 55.8%; 95% CI, 54.1%-57.4%) than negative (862/3576, 24.1%; 95% CI, 22.7%-25.5%) or neutral (720/3576, 20.1%; 95% CI, 18.9%-21.5%; Figure 1). Overall sentiment distributions differed between posts and comments (χ^2^ = 123.6, df = 2, *P* < .001). Across postcomment pairs (1 pair per comment), 64.5% (2306/3576; 95% CI, 62.9%-66.0%) were discordant; the most common discordant transition was negative post → positive comment (784/3576, 21.9%; 95% CI, 20.6%-23.3%; Figure 2), consistent with a strong culture of peer reassurance. Emotion tagging using the NRC Lexicon demonstrated higher normalized fear and sadness in posts and higher trust in comments (Supplemental Figure 1).

Medical terminology was common, with posts averaging 2.58 technical terms. However, users frequently conflated terms such as “ptosis,” “hooded eyes,” and “dermatochalasis,” and sometimes used “blepharoplasty” to refer to levator-based ptosis repair (Supplemental Table 1), reflecting persistent diagnostic ambiguity despite moderate clinical language familiarity.

### Thematic Analysis

A total of 500 Reddit entries were qualitatively analyzed. The analysis revealed 9 broad themes and 27 subthemes representing patient concerns and motivations, decision-making processes, and surgical reflections. Salience of recurring themes was described qualitatively by high-, medium-, or low-medium frequency estimates (Table 1). A joint display linking qualitative themes with predominant emotions observed in posts and comments is provided in Table 2.

**Table 1.** Frequency Estimates for Themes and Subthemes Within Reddit Posts Related to Eyelid Surgery

Frequency estimate	Theme	Definition	Example quote
High	Preoperative anxiety and hesitation	Emotional ambivalence or fear about committing to surgery or injections	“Something just doesn’t look right. I want to fix it, but I’m scared I’ll look worse.”
	Postoperative dissatisfaction or regret	Concerns about symmetry, new issues, or unmet expectations after a procedure	“I didn’t notice this roll until after surgery. I feel worse than before.”
	Functional vs cosmetic motivation	Blending of aesthetic goals with visual or eyelid-related dysfunction	“I can’t wear eyeliner anymore. It's not just about looks—it affects my confidence.”
	Peer support and validation	Emotional solidarity and normalizing of asymmetry, aging, or recovery experience	“You have beautiful eyes! I wouldn’t even notice if you hadn’t said anything.”
	Ambiguity in eyelid terminology	Confusion between ptosis, hooded eyes, dermatochalasis, and monolids	“I thought I had ptosis but maybe it's just a higher fold?”
	Difficulty identifying “normal”	Questioning whether droop, swelling, or asymmetry warrants concern or treatment	“Is it just age or should I be worried about this sagging crease?”
	BoNT hesitancy or misinformation	Fear of drooping, “frozen face,” or incorrect injection technique	“I’m scared Botox will make my brows heavier and my eyes more tired.”
	Temporary interventions	Eyelid tape, gua sha, makeup, and lighting as noninvasive coping or experimentation	“Sometimes my tape works and holds the crease all day. Other times it just folds again.”
	BoNT for eyebrow lift	Frequent discussion of using BoNT to lift the brow and reduce hooding	“Botox is the only thing that makes me look awake without surgery.”
	Eye asymmetry distress	Frustration or shame over 1 eye appearing more droopy, puffy, or creased	“Why does my left eye always look deeper set? It's all I can see in pictures.”
Medium	Cost as deterrent	High treatment costs deter users from pursuing surgery or BoNT	“I'd love to fix it, but $5,000 is not realistic for me right now.”
	surgeon trust and mismatch	Feeling dismissed or underserved by a provider's assessment	“She told me to get tox, but I wanted to know if it was drooping or swelling.”
	Overpromising or underexplaining	Perceived lack of informed consent about realistic outcomes	“I gave a glowing review, but now I feel like she didn’t warn me about this sagging.”
	Second opinions and vetting surgeons	Users seek aesthetic alignment and technical skill through reviews, photographs	“I spent weeks comparing before-and-after photos before choosing a clinic.”
	Medical concerns or diagnostic delay	Suspected underlying medical causes for asymmetry or ptosis	“Someone told me to get checked for keratoconus or a stroke. Should I be worried?”
	Misattribution to skin care products	Users wonder if eyelid changes are caused by creams, allergies, or sleep	“Could retinol be making my lid puffy or did I just age overnight?”
	Social Media influence	Beauty ideals and dissatisfaction driven by filtered or curated online content	“When I see those TikTok girls with clean lids, I feel like mine are melting.”
Low-Medium	Cultural and ethnic norms	Awareness that aesthetic standards for eyelids vary by ethnicity or region	“In my culture, hooded eyes are normal and not seen as something to fix.”
	Insurance navigation confusion	Uncertainty about coverage for functional problems vs cosmetic requests	“How bad does it have to be for insurance to pay for ptosis repair?”

BoNT, botulinum toxin.

**Table 2.** Joint Display Linking Qualitative Themes to Predominant Emotions in Posts and Comments

Theme	Predominant emotions in posts (NRC/transformer)	Predominant emotions in comments (NRC/transformer)	Interpretive linkage
Psychosocial impact and self-perception	Sadness, fear	Trust, joy	Self-esteem distress in posts is often met with reassurance and normalization in comments.
Surgical expectations, fear, and regret	Fear, anticipation; sadness/anger (regret)	Trust, anticipation	Preoperative dread and early recovery uncertainty are commonly answered with “wait-and-see” reassurance.
Clinical communication and surgeon selection	Anticipation, fear; anger (dismissal)	Trust, anticipation	Posts about surgeon choice and perceived dismissal elicit peer guidance and crowd-sourced vetting.
Navigating nonsurgical interventions	Anticipation, fear	Trust, anticipation	Experimentation with Botox/drops/creams is framed as low stakes; comments provide practical tips and caution.
Functional vs aesthetic motivation	Anticipation, fear	Trust, anticipation	Functional symptoms are often narrated through an aesthetic lens; comments encourage evaluation and expectation setting.
Peer support and normalization	Sadness, fear	Trust, joy	Community responses frequently reframe concerns as common and manageable.
Systemic and financial barriers	Anger, sadness	Trust, anticipation	Cost/insurance stress is common; comments share strategies and normalize tradeoffs.
Cultural and societal influences	Anticipation, sadness	Trust, anticipation	Posts reference culturally inflected aesthetic ideals; comments discuss norms and medical tourism.
Terminological confusion and diagnostic uncertainty	Fear, anticipation	Trust, anticipation	Diagnostic ambiguity in posts prompts corrective guidance, reassurance, and referral recommendations.

NRC, National Research Council Emotion Lexicon.

#### Theme 1: Psychosocial Impact and Self-Perception

Many Reddit users described using eyelid surgery as a tool to feel more “awake” or more confident. Such commentary suggests that patients seek surgery not just for appearance but as a psychological “fix” for self-esteem.

**Eyelid asymmetry distress**: Reddit users expressed disproportionate concern over subtle asymmetries, particularly uneven eyelids. One user commented, “My left eye looks so weird in pictures, it's ruining my confidence,” suggesting that the perception of asymmetry in photographs, makeup, or lighting—especially in the context of body dysmorphia or perfectionism—may override functional satisfaction.

**Obsession with minor flaws**: Users focused on subtle changes, such as quadruple lids or slight droops. One user, referring to their eyelid fold, stated that “no one else notices,” but they “can’t stop seeing the fold getting higher and looser.” These comments suggest a preoccupation with differences, some as small as 1 to 2 mm, that may be considered within normal limits by surgeons.

**Makeup as a coping strategy**: Eyeliner, lashes, and shadow were often described as tools to conceal or reshape appearance. One individual stated, “If I do my liner at a certain angle and squint, it hides the droop a little,” demonstrating how makeup helps patients mitigate worries about perceived imperfections.

**Aging and self-perception**: Patients expressed anxiety and grief over appearance changes related to age. One Reddit user described feeling “like a sad, dry, pale, droopy, middle-aged f*ck.” When aging threatens individuals' perception of their own beauty and worth, surgery may be seen as a restorative intervention.

#### Theme 2: Surgical Expectations, Fear, and Regret

Reddit users frequently expressed emotional distress before and after eyelid surgery. Ongoing ambivalence regarding the pursuit or result of surgical intervention was also a recurring topic of discussion.

**Preoperative anxiety**: Many users expressed preoperative anxiety regarding fear of poor outcomes, asymmetry, or irreversible change. One user explained, “I’ve waited a year for ptosis surgery but now I’m scared it’ll make things worse,” indicating fear of regret despite desired change.

**Postoperative disappointment**: Some express regret following blepharoplasty or ptosis repair. One user described dissatisfaction with their surgery: “Now my brow sags and I look worse than before—wish I never touched it.” Perceived poor surgical outcomes or new postoperative aesthetic concerns may impact patient self-confidence.

**Ambivalence about surgery**: Users also expressed hesitancy about eyelid surgery, even after long periods of planning. Despite 1 patient having “waited years for ptosis surgery,” they remained “unsure” about proceeding, revealing tension between fear and desire for change.

#### Theme 3: Clinical Communication and Surgeon Selection

Patients are increasingly choosing a surgeon based on Reddit recommendations, influencer videos, or TikTok before and after photographs—often placing more emphasis on these sources than board certification or professional reputation. These results indicate that the process of surgeon selection is driven more by anecdotal reviews and social media than by credentials.

**Surgeon trust and mismatch**: Patient concerns about being dismissed or misunderstood by providers were frequently discussed. One user referred to their surgeon and remarked, “She told me to get tox, but I wanted to know if it was drooping or swelling.” This disparity between patients' concerns surgeons' responses may reduce trust and cause uncertainty about pursuing surgical intervention.

**Overpromising or underexplaining**: Many users express dissatisfaction after surgery not because of complications but because the results did not meet their aesthetic expectations—particularly regarding symmetry, eye shape, or lash visibility. One user offered a postoperative reflection, “I gave a glowing review, but now I feel like [the surgeon] didn’t warn me about this sagging.” Surgeons tend to evaluate success based on eyelid height, margin–reflex distance, or functional improvement, but patients are focused on fine-grained, aesthetic outcomes.

**Second opinions and “Do-It-Yourself (DIY)” vetting**: Patients report researching and seeking out “aesthetic sense” in surgeons. A user described spending “weeks comparing before-and-after photos before choosing a clinic,” emphasizing the importance of personal investigation and goal alignment between the patient and surgeon.

#### Theme 4: Navigating Nonsurgical Interventions

Patients often explore nonsurgical methods to address concerns about their eyelid appearance. Reddit discussions reveal eye product and botulinum toxin (BoNT) experimentation in addition to “DIY” fixes.

**Trial and error with products**: Reddit users discuss testing various creams, eyelid tapes, and serums with mixed results. Some users express frustration with inconsistent or disappointing results, stating, “I’ve been using ginseng eye serum for months—no change at all.”

**Botulinum toxin hesitation and curiosity**: Although many patients report considering BoNT (usually referred to as “Botox”) treatment, many also fear potential side effects, especially eyelid ptosis. One user admitted, “I want to try it, but I’m terrified I’ll end up with one droopy eye for months,” revealing a common hesitancy deterring patients from trying BoNT.

**DIY and online advice seeking**: Reddit serves as a central hub for users to share personal experiences, tutorials, hacks, and “inner corner” tricks. One user suggested, “Try doing your eyeliner outline with eyes open—helped my hooded lids so much!” This supportive exchange fosters a sense of community among patients.

#### Theme 5: Functional vs Aesthetic Motivation

Compared with aesthetic concerns, few Reddit users mention visual field restriction or eyelid function. Even among patients eligible for insurance-covered procedures, the conversation reveals primarily aesthetic motivations. Reddit discussions focused on diagnostic uncertainty, appearance reflecting lifestyle changes, and nonsurgical options to address eyelid concerns.

**Uncertainty about medical diagnosis**: There was significant confusion among Reddit users regarding whether their eyelid changes were medical issues—such as ptosis—or associated with typical variation or aging. One user asked, “Is it droopy? Swollen? Or just uneven eyes? I honestly can’t tell.” This diagnostic uncertainty often led patients to seek reassurance and peer opinions online.

**Aging and fatigue as triggers**: Patients frequently attributed eyelid sagging or asymmetry to motherhood, weight loss, or aging. One user reported, “I used to have even lids but ever since I had my baby, they’ve drooped so much.” Life changes can heighten self-awareness and concerns about appearance.

**Desire for “natural” correction**: Many users prefer nonsurgical approaches including creams, massage, or facial yoga. For example, a user asked, “Is there anything besides blepharoplasty that can fix this? I want to avoid surgery at all costs.” This preference reflects patient apprehension surrounding surgical intervention and the conception of surgery as “unnatural.”

#### Theme 6: Peer Support and Normalization

Patients seek support from Reddit's online community, using the platform to share experiences, exchange empathy, and gain reassurance from others.

**Empathic community support**: Users affirm each other's concerns and offer emotional support through compassionate and validating messages. One user reassured another, “You’re the only one who notices it, I promise. You look beautiful.” These peer-to-peer exchanges foster a sense of belonging and reduce feelings of isolation.

**Normalization of asymmetry**: Reddit community members remind others that facial asymmetry is common and expected. Assurances such as “Everyone has uneven eyes—it's human. Don’t be so hard on yourself,” normalizes perceived imperfections and combats self-criticism.

**Advice from shared experience**: Many patients share personal stories of treatments or interventions that worked, or did not work, to help guide others. One user shared, “I did upper bleph, and honestly, it made a big difference. No regrets.” Patients look to each other for knowledge and advice to inform their treatment decisions.

#### Theme 7: Systemic and Financial Barriers

Reddit users lament systemic and financial barriers to accessing surgical and medical treatments. User commentary indicates that high costs and ambiguity in insurance coverage limit access to and utilization of care.

**Cost as deterrent**: The high cost of surgery or BoNT is a significant barrier, preventing patients from accessing treatment or causing delayed decision making. One user remarked, “I'd love to fix it, but $5000 is not realistic for me right now,” reflecting that even when patients are motivated to seek care, financial obstacles may be insurmountable.

**Insurance navigation confusion**: Many users expressed confusion about what qualifies as “functional” or “cosmetic.” Questions such as “How bad does it have to be for insurance to pay for ptosis repair?” reflect uncertainty about medical necessity and insurance coverage criteria for eyelid surgery.

#### Theme 8: Cultural and Societal Influences

Reddit discourse revealed that cultural and media-portrayed ideals of beauty heavily influence patients' goals and shape expectations far more than functional issues.

**Ethnic and regional norms**: Reddit entries show recognition among users that eyelid standards vary by culture. One user mentioned, “In my culture, hooded eyes are normal and not seen as something to fix.” For Asian patients especially, there is frequent discussion of double eyelids, “doll eyes,” or “cat eyes,” often referencing Korean or Chinese beauty standards.

**Social media comparisons**: Reddit entries suggested that contemporary beauty ideals are significantly shaped by influencers and filters. One user commented, “When I see those TikTok girls with clean lids, I feel like mine are melting,” suggesting frequent appearance-related self-comparison to social media figures and consequently heightened aesthetic awareness and focus on one's own flaws.

**Normalization of cosmetic intervention**: Online discourse suggests that surgery is becoming a mainstream aesthetic decision. For example, 1 user reported, “I’m 20 and already thinking about eyelid surgery,” indicating an increasingly normalized view of cosmetic intervention and early internalization of beauty standards.

#### Theme 9: Terminological Confusion and Diagnostic Uncertainty

Reddit users frequently misused medical terms and expressed uncertainty related to the diagnosis of eyelid conditions.

**Ambiguity in eyelid terms**: Many patients do not understand the difference between ptosis, hooded eyelids, dermatochalasis, or double eyelids. For example, 1 user inquired, “I thought I had ptosis but maybe it's just a higher fold?” This confusion affects patients' ability to choose the right procedure or consult the right specialist.

**Difficulty identifying “normal”**: Patients have difficulty distinguishing between natural asymmetry and pathology. One individual asked, “Is it just age or should I be worried about this sagging crease?” This indicates patients' struggle to assess whether changes in their appearance are problems that warrant intervention.

## DISCUSSION

This cross-sectional, mixed-methods analysis of Reddit conversations offers insights into how patients discuss and experience eyelid surgery, revealing emotional, cultural, and informational themes that complement traditional patient-reported outcomes. Posts showed mixed polarity, whereas comments skewed positive, consistent with peer reassurance. Fear and sadness were more prominent in posts, whereas trust (and anticipation) was more prominent in comments. Thematic analysis uncovered 9 interrelated themes. Prominent among them were preoperative anxiety; postoperative disappointment despite technically acceptable measurements; and persistent confusion over terms such as ptosis, hooded lids, dermatochalasis, and “double eyelids.” Participants frequently experimented with nonsurgical approaches (creams, tapes, and BoNT) and described cost and insurance ambiguity as barriers. Cultural frames—particularly East Asian beauty ideals emphasizing crease formation and specific eye shapes—recurrently shaped what outcomes patients hoped to achieve. These narratives foreground psychosocial motives at least as much as, if not more, than functional concerns.

Although many individual concerns are familiar in clinic, Reddit adds measurable insight into how patients narrate these concerns outside structured PROM framing, quantifies the high rate of postcomment discordance consistent with peer reassurance, and surfaces recurrent terminology mix-ups that may redirect care-seeking.

### Language, Literacy, and Diagnostic Uncertainty

Although posts contained a moderate density of medical terminology (mean 2.58 technical terms), usage was often imprecise. Patients regularly misapplied labels (eg, conflating dermatochalasis with ptosis), a gap that can redirect care-seeking (eg, toward blepharoplasty when levator repair is indicated) or seed postoperative regret when aesthetic expectations are not aligned with anatomic constraints. These findings suggest that preoperative encounters benefit from early, visual, and vocabulary-anchored education that distinguishes diagnoses, procedures, and realistic aesthetic endpoints.

### Aesthetics, Asymmetry, and the Limits of “Normal”

Across threads, aesthetic success was framed less by anatomical and surgical measurements and more by image-based appraisals of lash show, crease shape, “almond/cat-eye” contours, and perceived symmetry—standards often influenced by filtered photographs and intracultural ideals. Surgeon reassurances that “some asymmetry is normal” often failed to resolve distress; in select patients, perfectionistic attention to minute differences may predict dissatisfaction even after functionally successful outcomes. Incorporating structured discussion of asymmetry tolerance and, when indicated, screening for body image concerns may improve shared decision making.

The psychological weight patients place on procedures such as eyelid surgery may be underestimated. Although not pathological, these desires are often emotionally charged, and poor results can have dramatic impacts on self-image. Surgeons may consider preoperative screening for emotional readiness and assessment of concern over asymmetry or body image, providing reassurance where appropriate, and using image-based goal alignment to promote shared understanding. Surgeons should also consider potential ethical challenges associated with social media visibility. Online testimonials and aesthetic content shape patients' expectations and may be more highly emphasized than clinical expertise. Building a digital presence focused on authentic patient education and transparent before and after photographs may create trust and counter misinformation.

### Internet and Social Media Influence

Previous studies examining online patient communication regarding oculoplastic surgeries have focused on content more than sentiment and have found that social media platforms facilitate discussions that primarily include advice, before and after photographs, procedure costs, and general support and encouragement.^19^ Notably, researchers have also found that patient posts with positive surgical outcomes are significantly more upvoted and prominent than those with negative or failed outcomes, introducing a potential source of selection bias in social media conversations.^20^ Although other medical specialties, including neurosurgery and otolaryngology, have begun to explore online patient sentiment regarding surgical outcomes, to the best of our knowledge, this is the first report to use Reddit posts to quantify specific emotions surrounding oculoplastic surgery.^9-12^

Finally, social media users frequently described surgeon selection through Reddit threads and influencer videos rather than through credentials. This ecosystem elevates visibility and testimonials, risks overpromising through edited imagery, and can nudge patients toward procedures misaligned with their anatomy or goals. A proactive, ethical digital presence—centered on educational visuals, procedure indications/limitations, and frank discussions of variability—may counter misinformation, enhance physician–patient trust, and improve patient outcomes and satisfaction.

### Clinical Implications

These practice suggestions are interpretive takeaways derived from observed discourse patterns in this dataset and are intended to inform counseling and shared decision making rather than serve as prescriptive standards.

- **Start with a shared vocabulary.** Open consults by differentiating ptosis, dermatochalasis, hooding, and double-eyelid anatomy using annotated photographs or mirror-based review; verify the patient's working diagnosis and what each procedure can—and cannot—change.
- **Align on aesthetics, not just metrics.** Invite patients to specify their “ideal look” with reference images; translate those into anatomical feasibilities and tradeoffs (crease height, lash show, lateral flare, and brow–lid interplay). Set ranges rather than absolutes for symmetry and shape.
- **Normalize variability and probe for distress.** Discuss typical asymmetry and photography effects; when 1 to 2 mm differences drive disproportionate concern, consider brief screening for body image vulnerability and—when appropriate—collaboration with mental health professionals.
- **Address the “psychological fix.”** Many patients seek to look less tired or more confident; validate these motives while clarifying that aesthetic change may not resolve broader self-esteem concerns. Frame outcomes as improvements with variability, not certainties.
- **Acknowledge the social media context.** Ask where expectations came from; review how filters, pose, and lighting influence “after” images; explain how selection bias and algorithmic visibility can skew perceived norms. Offer your own transparent gallery and educational content.
- **Integrate functional and cosmetic aims.** Even when visual field loss or lash–corneal touch is present, invite patients to articulate aesthetic hopes so functional plans do not inadvertently disappoint cosmetic expectations.
- **Clarify costs and coverage early.** Demystify what insurers consider “functional,” outline documentation requirements (visual fields and photographs), and present staged or nonsurgical options when appropriate.

### Limitations and Future Research

There are several limitations of this analysis. We focused solely on text included in Reddit posts and did not account for visual content such as photographs, which are particularly relevant in discussions of aesthetic outcomes. Additionally, we did not examine the relationship between emotional tone and time since or leading up to surgery, nor did we stratify posts by demographics, largely because of user anonymity. Furthermore, upvotes, which reflect endorsement from other users, were not considered. Importantly, Reddit users are not necessarily representative of the general eyelid surgery population: Reddit tends to skew toward younger, digitally engaged users, and participation is self-selected (individuals with high concern, uncertainty, or strong outcomes may be more likely to post). Sampling Reddit discussions may also inherently overrepresent patients seeking peer reassurance (eg, r/PlasticSurgery and r/HoodedEyes) and underrepresent patients who do not use social media or who primarily engage with offline support networks. Moreover, because we prespecified 8 subreddits, eyelid surgery discussions occurring in other communities may have been missed, and theme prevalence may differ in subreddits with different norms, demographics, or moderation practices. Finally, comments were sampled using Reddit's default “Best” ranking algorithm rather than random sampling, which may preferentially surface supportive or high-engagement responses.

Future studies may include other social media platforms such as TikTok and YouTube, which attract younger demographics and emphasize video-based communication.^21^ However, these platforms will introduce new methodological challenges, including identifiable users, account visibility bias, and sponsorship impact on the tone and reach of posts. Although these factors could skew emotional sentiment or introduce selection bias, they also provide an opportunity to analyze the emotional and psychological impact of highly visible aesthetic content that may influence the perceptions and expectations of large patient populations.

## CONCLUSIONS

Reddit discussions about eyelid surgery reveal patients who use clinical terms yet are uncertain about diagnoses; who often judge the success of a procedure by culturally inflected, photo-salient aesthetics; and who rely on peer communities to soothe fears and validate decisions. Centering this “patient voice” in clinic—through early terminology alignment, image-based goal setting, explicit discussion of asymmetry and variability, and ethically transparent online education—may inform counseling approaches aimed at reducing avoidable mismatch between expectations and outcomes, which should be evaluated in prospective clinical studies.

## Supplemental Material

This article contains supplemental material located online at https://doi.org/10.1093/asjof/ojag050.

## Supplementary Material

ojag050_Supplementary_Data
