# Supplementary material for: Decoding the Digital Discourse: A Thematic and Sentiment Analysis of Reddit Posts on Eyelid Surgery
Source: Aesthet Surg J Open Forum. 2026 Mar 18;8:ojag050. doi: 10.1093/asjof/ojag050 (PMC13089458; doi:10.1093/asjof/ojag050)
Supplement: ojag050_Supplementary_Data [file ojag050_supplementary_data.zip › Supplemental Figure Legend.docx]

**Supplemental Figure Legend**

**Supplemental Figure 1**. Normalized average emotion intensity scores for posts and comments, derived using the NRC Emotion Lexicon.
