# Supplementary material for: Decoding the Digital Discourse: A Thematic and Sentiment Analysis of Reddit Posts on Eyelid Surgery
Source: Aesthet Surg J Open Forum. 2026 Mar 18;8:ojag050. doi: 10.1093/asjof/ojag050 (PMC13089458; doi:10.1093/asjof/ojag050)
Supplement: ojag050_Supplementary_Data [file ojag050_supplementary_data.zip › Supplemental Table 1.docx]

**Supplemental Table 1**. Examples of Terminology Confusion (Paraphrased; Labeled By Subreddit and Month/Year)

| Confusion Type | Subreddit | Month-Year | Paraphrased example |
| --- | --- | --- | --- |
| Ptosis vs hooding/dermatochalasis | 30PlusSkinCare | Jun 2024 | User asks whether “ptosis” is actually hooded lids/excess skin or normal asymmetry, and what intervention (if any) would address it. |
| Ptosis vs hooding/dermatochalasis | HoodedEyes | Apr 2025 | User describes uneven lids and labels it ptosis, but simultaneously frames it as “hooded eyes” and asks what it “really is.” |
| Blepharoplasty vs ptosis repair | PlasticSurgery | Apr 2025 | User treats blepharoplasty and ptosis repair as interchangeable and asks which one “fixes droopy eyelids.” |
| Blepharoplasty vs ptosis repair | PlasticSurgery | May 2025 | User asks whether levator-based ptosis repair and skin-removal blepharoplasty are essentially the same operation for eyelid droop. |
| Brow ptosis vs eyelid ptosis | 30PlusSkinCare | Mar 2025 | User is unsure whether heaviness is from brow descent vs eyelid ptosis and asks if brow lift/Botox vs ptosis repair/bleph is appropriate. |
| Ptosis vs neurologic emergency | AskDocs | Jun 2024 | User reports acute ptosis with headache/neurologic concern and asks whether this could represent stroke/aneurysm/cranial nerve palsy and where to seek care. |
| Ptosis as proxy for myasthenia/Horner | Ophthalmology | Dec 2024 | User treats ptosis as a marker of myasthenia gravis or Horner syndrome and asks about diagnostic testing and workup. |
| Ptosis vs strabismus/lazy eye | PlasticSurgery | Dec 2024 | User describes eye misalignment (lazy eye/strabismus) plus eyelid asymmetry and asks whether ptosis surgery would correct overall appearance. |
| Specialist role confusion | 30PlusSkinCare | Feb 2025 | User is unsure whether to consult oculoplastics vs general plastics vs ophthalmology vs neurology for eyelid droop. |
| Medication-related confusion | 30PlusSkinCare | Jul 2024 | User links eyelid droop to topical drops/serums (e.g., oxymetazoline/prostaglandin analogues) and asks whether this is “ptosis” or a medication effect. |
| Hering’s law / contralateral droop | cosmeticsurgery | Apr 2025 | User references Hering’s law to explain contralateral eyelid droop after correction and asks whether it is expected and reversible. |
